# Formulation‐Dependent Cytotoxic Effects of Commercial and Analytical‐Grade Mancozeb in C6 Glial Cells

**DOI:** 10.1002/jat.70214

**Published:** 2026-04-20

**Authors:** Lavínia Lazzarotti, Felipe Vanz, Karina Giacomini Varela, Viviane Glaser, Diego de Carvalho, Aline Pertile Remor

**Affiliations:** ^1^ Graduação em Farmácia Universidade do Oeste de Santa Catarina (UNOESC) Campus de Joaçaba SC Brazil; ^2^ Programa de Pós‐Graduação em Biociências e Saúde (PPGBS), Área de Ciências da Vida e Saúde, Universidade do Oeste de Santa Catarina (UNOESC) Campus de Joaçaba SC Brazil; ^3^ Laboratório de Biologia Celular, Centro de Ciências Rurais, Coordenadoria Especial de Ciências Biológicas e Agronômicas Universidade Federal de Santa Catarina (UFSC) Campus de Curitibanos SC Brazil

**Keywords:** cytotoxicity, mancozeb, occupational and human exposure, oxidative stress, pesticide formulations

## Abstract

Mancozeb (MZ) is a widely used dithiocarbamate fungicide composed of manganese (Mn) and zinc (Zn) complexes. Increasing concern has been raised regarding its occupational and human health relevance, particularly due to its neurotoxic potential associated with Mn‐related cellular dysfunction. Despite being marketed exclusively as commercial formulations, most toxicological investigations rely on analytical‐grade MZ, thereby neglecting the potential contribution of coformulants to toxicity. In this study, we comparatively evaluated the effects of commercial and analytical‐grade MZ in C6 astroglioma cells, focusing on cell viability and intracellular reactive oxygen species (ROS) generation. Cells were initially exposed to increasing concentrations of the commercial formulation (1–500 μM) for 1, 3, 6, and 24 h to characterize time‐ and concentration‐dependent cytotoxicity. Based on this screening, analytical‐grade MZ was subsequently evaluated at selected concentrations corresponding to the onset of cytotoxic effects. Comparative analyses were then performed after 6 h of exposure to a shared concentration range (10–50 μM) to assess formulation‐specific effects. Both commercial and analytical‐grade MZ induced significant reductions in cell viability in a time‐ and concentration‐dependent manner. Although both formulations significantly decreased cell viability starting at 20 μM after 6 h relative to control, a concentration‐dependent difference between formulations was observed. At 50 μM, the commercial formulation produced a greater reduction in cell viability (~60%) compared to the analytical‐grade MZ (~40%), both relative to controls; this difference between formulations was also supported by the two‐way ANOVA. In contrast, intracellular ROS levels increased to a similar extent following exposure to both formulations, with no significant differences observed between them. These findings indicate that the enhanced cytotoxicity induced by commercial MZ is not solely explained by oxidative stress, suggesting the contribution of formulation‐specific components. Overall, this study underscores the importance of evaluating complete pesticide formulations to achieve a more realistic assessment of toxicological hazard under occupational and human health exposure scenarios.

## Abstract 2

Commercial formulations of mancozeb may exhibit greater toxicity than the active ingredient alone. Here, we show that commercial mancozeb induces higher cytotoxic effects in C6 glial cells than analytical‐grade MZ, despite similar increases in ROS. These findings indicate that enhanced toxicity is not solely driven by oxidative stress and may involve coformulants and formulation‐dependent changes in solubility and metal ion availability. This study highlights the importance of evaluating complete formulations to improve toxicological risk assessment under real‐world exposure conditions.

## 1 Introduction

Mancozeb (MZ) is a broad‐spectrum fungicide composed of manganese (Mn) and zinc (Zn) atoms coordinated with ethylene bisdithiocarbamate (Costa‐Silva et al. 2018). Initially synthesized in the 1940s for seed treatment, MZ has remained in extensive agricultural and industrial use for nearly nine decades, due to its high efficacy and cost‐effectiveness (Runkle et al. 2017). Like most pesticides, it is commercially available in several formulations, including dust, liquid, dispersible granules, and wettable powder. However, increasing concerns have been raised regarding its environmental impact, particularly with respect to the contamination of aquatic ecosystems, with potential ecological consequences and implications for human exposure through agricultural and occupational settings (de van Wenl Joo et al. 2016).

In addition to environmental concerns, occupational exposure to MZ has been associated with a range of adverse health effects, including endocrine disruption, behavioral alterations, genotoxicity, and neurotoxicity (Balaji et al. 2014; Pirozzi et al. 2016; Srivastava et al. 2016). Although the precise molecular mechanisms underlying these effects are not fully elucidated, experimental evidence suggests a central role for increased ROS production and mitochondrial dysfunction, largely attributed to Mn present in the MZ molecule (Tsang and Trombetta 2007; Costa‐Silva et al. 2018). Although Mn is an essential trace element, excessive accumulation, particularly in the central nervous system (CNS), may lead to a Parkinsonian‐like syndrome known as manganism (Rovetta et al. 2007).

Due to its recognized reproductive toxicity and endocrine‐disrupting properties, the use of MZ was banned in the European Union in 2021 (European Food Safety Authority et al. 2020). Nevertheless, MZ continues to be widely employed in agriculture in several countries (Panis et al. 2022). In Brazil, the 2023 Annual Report on the Production, Import, Export, and Sales of Pesticides ranked MZ as the second most sold active ingredient, surpassed only by glyphosate and its salts (Brasil 2024). This persistent and large‐scale use underscores ongoing concerns regarding human exposure, particularly in agricultural and occupational contexts.

Importantly, pesticide formulations, including those containing MZ, consist of mixtures of the declared active ingredient and various coformulants, many of which are often classified as inert and protected as confidential business information (Defarge et al. 2016; UPL Brasil 2024). Regulatory toxicological assessments, however, typically focus on isolated active ingredients. Several studies have demonstrated that pesticide formulations can be more toxic to human cells than the corresponding active compounds alone (Benachour and Séralini 2009; Ferguson et al. 2022; Nagy et al. 2021; Nikoloff et al. 2013; Simasotchi et al. 2021). Coformulants are not biologically inert; rather, they are designed to enhance the stability, bioavailability, and cellular uptake of active ingredients, potentially amplifying toxic responses. This highlights the critical need to investigate the toxicological effects of complete pesticide formulations rather than individual active substances, particularly in the context of applied and human toxicology (Mesnage et al. 2013).

Astrocytes represent the predominant glial cell population in the CNS and play essential roles in maintaining brain homeostasis, including metabolic support to neurons, regulation of synaptic activity, maintenance of ion and neurotransmitter balance, and responses to injury and neurotoxic insults (Cotto et al. 2019). The C6 astroglioma cell line, originally derived from a rat brain tumor induced by *N*‐nitrosomethylurea (Benda et al. 1968), exhibits astrocytic characteristics, including high expression of glial fibrillary acidic protein (GFAP) and has been widely used as an in vitro model to investigate the effects of neurotoxic compounds (Haghighat et al. 2000; Haghighat and McCandless 1997). Therefore, C6 cells represent a suitable experimental model to investigate mechanisms of pesticide‐induced neurotoxicity relevant to astrocyte biology.

Given the sensitivity of the CNS to Mn‐induced toxicity and considering that MZ is marketed as complex commercial formulations rather than as a pure compound, this study aimed to compare the toxicological effects of commercial and analytical‐grade MZ in C6 astroglioma cells, as an in vitro model relevant to applied and human toxicology, focusing on cell viability and oxidative stress‐related outcomes.

## 2 Material and Methods

### 2.1 Chemicals

All chemicals used for cell culture were of analytical‐grade or the highest pharmaceutical grade available. Commercial MZ (manganese/zinc ethylene bisdithiocarbamate) was obtained as Unizeb Gold (UPL do Brasil, Indústria e Comércio de Insumos Agropecuários S.A.), a water‐dispersible granule (WG) formulation containing 750 g/kg (75% w/w) of the active ingredient, and analytical‐grade MZ (Pestanal) was purchased from Sigma‐Aldrich, USA.

### 2.2 C6 Cell Line Maintenance

C6 rat glioma cells (ATCC CCL‐107) were obtained from the American Type Culture Collection (Rockville, MD, USA) and cultured in flasks containing Dulbecco's Modified Eagle's Medium (DMEM) supplemented with 5% (v/v) fetal bovine serum (FBS) and an antibiotic–antimycotic solution (100x), diluted 1:100 to obtain final concentrations of penicillin (100 U/mL), streptomycin (100 μg/mL), and amphotericin B (0.25 μg/mL). Amphotericin B was used as a standard antifungal agent and was present in all experimental conditions, including controls. Cultures were maintained at 37°C with a minimum of 95% relative humidity and in an atmosphere of 5% CO_2_. Cells were trypsinized using 0.05% trypsin/ethylene‐diaminetetraacetic acid (EDTA) and seeded in plates of appropriate sizes. Treatments were initiated when cells reached 80% confluence.

### 2.3 Experimental Protocol

To evaluate time‐ and concentration‐dependent effects of MZ toxicity, C6 cells were first exposed to the commercial formulation at concentrations ranging from 1 to 500 μM for 1, 3, 6, or 24 h, followed by assessment of cell viability. Based on this initial screening, analytical‐grade MZ was subsequently tested at the concentrations considered most informative for each time point, corresponding to the concentration immediately below the onset of cytotoxicity and the first concentration producing a significant reduction in viability in the commercial formulation.

Then, to directly compare the toxicological profiles of commercial and analytical‐grade MZ, cell viability and intracellular ROS generation were evaluated after 6 h of exposure to a shared concentration range of 10–50 μM. MZ formulations were initially dissolved in dimethyl sulfoxide (DMSO), a widely used solvent for poorly water‐soluble compounds in cell culture systems, to prepare stock solutions. The final concentration of DMSO in culture medium did not exceed 0.01% in any experimental condition, and this concentration was maintained constant across all groups, including controls. Corresponding vehicle controls were included in all experiments, and cell viability results were expressed relative to the vehicle control (100%).

### 2.4 Cell Viability Assay

Cell viability was determined using the MTT reduction assay (3‐(4,5‐dimethylthialzolyl‐2‐yl)‐2,5‐diphenyltetrazolium bromide), as described by Mosmann (1983). This assay is based on the reduction of MTT by mitochondrial dehydrogenases in viable cells, resulting in the formation of insoluble formazan crystals, a purple‐colored compound measurable spectrophotometrically.

For the assay, cells were seeded in 96‐well plates and, following the treatment protocol, incubated with MTT solution (0.5 mg/mL) for 30 min at 37°C. Afterward, formazan crystals were solubilized in 200 μL of DMSO, and absorbance was measured at 540 nm using a BioTek plate reader. Results were expressed as a percentage of the control, using the average absorbance of each group. All experiments were performed three times, in triplicate, independently.

### 2.5 Intracellular ROS Production Assay

Intracellular ROS production was assessed using 5‐(and‐6)‐carboxy‐2′,7′‐dichlorofluorescein diacetate (carboxy‐H2DCFDA, Image‐iT Live Green, Molecular Probes, Carlsbad, CA, USA). C6 cells were seeded in black 96‐well plates, and, after the treatment protocol, culture medium was removed, and cells were washed with Hank's Buffered Saline Solution (HBSS). Cells were then incubated with 10‐μM carboxy‐H_2_DCFDA for 30 min at 37°C. Following intracellular deacetylation by esterases, the probe reacts with ROS to generate the fluorescent product carboxy‐DCF (Ali et al. 1992). Fluorescence was measured at 485 (excitation) and 538 nm (emission) using a plate reader. Results were expressed as a percentage of the control, using the mean fluorescence intensity of each group. All experiments were performed three times, in triplicate, independently. Hydrogen peroxide (H_2_O_2_) at 100 μM was used as a positive control for the ROS detection assay and was not included as an experimental group.

### 2.6 Protein Determination

Cell homogenates were used for protein quantification, which was determined using the method of Lowry et al. (1951), with bovine serum albumin as the standard curve.

### 2.7 Statistical Analysis

Results were expressed as mean ± standard error of the mean (SEM). Data were analyzed by one‐way ANOVA for independent group comparisons within each formulation (1, 3, 6, and 24 h) and two‐way ANOVA to compare formulation‐dependent effects on cell viability and intracellular ROS generation after 6 h of exposure, followed by Tukey's post hoc test when *P* was significant. The statistical outcomes presented in the results section are based on these ANOVA models and their corresponding interaction and main effects. A *p* < 0.05 was considered statistically significant. Only statistically significant *P* values are reported in the text. Statistical analyses were performed using Statistical Package for the Social Sciences (SPSS, version 16.0 for Windows) and/or GraphPad Prism 10. All graphs were plotted using GraphPad Prism 10.

## 3 Results

**FIGURE 1:**
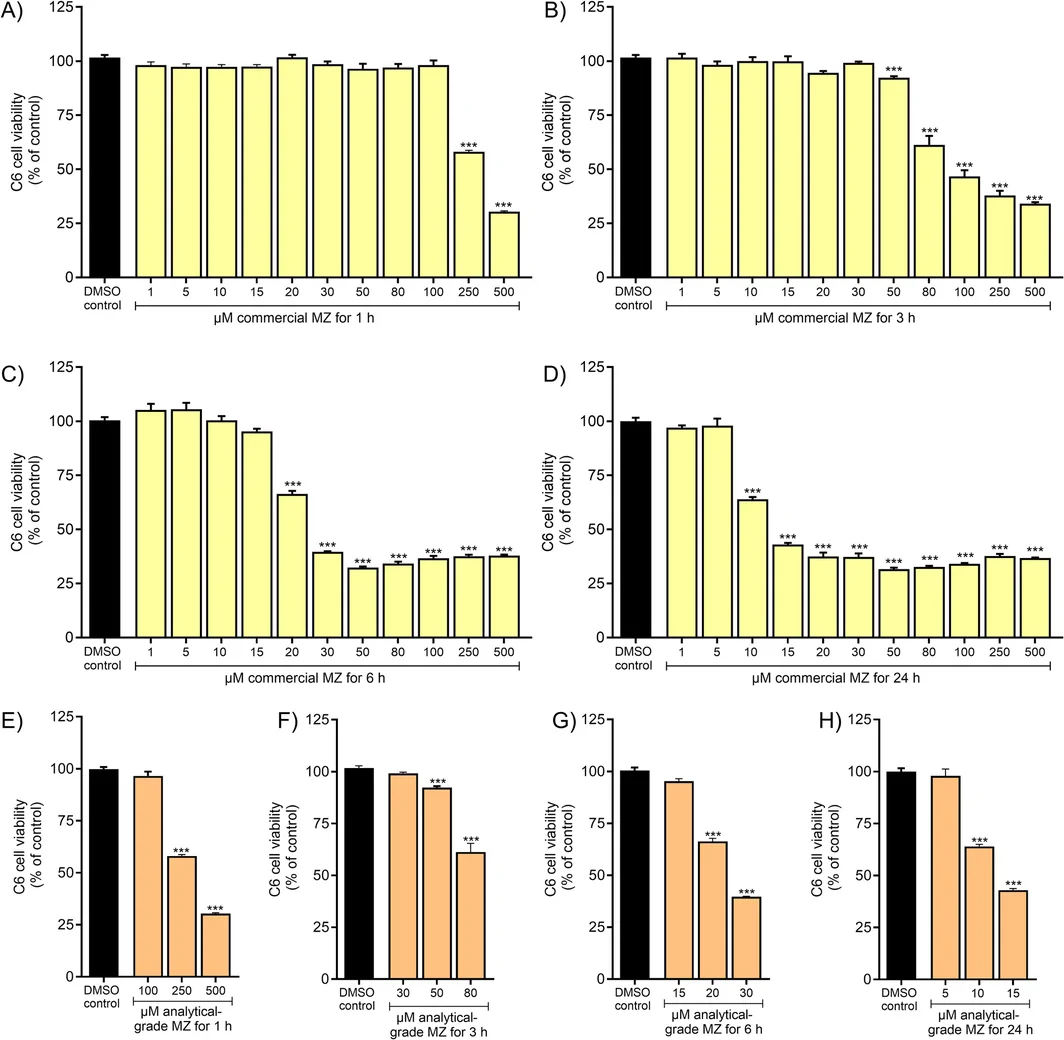
Effects of commercial and analytical‐grade Mancozeb (MZ) on C6 cell viability. C6 cells were incubated with increasing concentrations of commercial MZ (1–500 μM) for 1 h (A), 3 h (B), 6 h (C) and 24 h (D). Based on the commercial formulation screening, analytical‐grade MZ was evaluated at selected concentrations corresponding to the concentration immediately below the onset of cytotoxicity and the first concentration producing a significant reduction in viability at each time point: 1 h (E), 3 h (F), 6 h (G), and 24 h (H). Data are presented as mean ± SEM and expressed as a percentage of the control group. ***p < 0.001 compared with the DMSO control group (one‐way ANOVA followed by Tukey's post hoc test).

In order to evaluate the toxic effects of MZ on C6 cell viability, cells were initially incubated with increasing concentrations of the commercial formulation (1–500 μM) for 1, 3, 6, or 24 h (Figure 1A–D). Based on these findings, analytical‐grade MZ was then evaluated at selected concentrations corresponding to the concentration immediately below the onset of cytotoxicity and the first concentration that significantly reduced viability at each time point (Figure 1E–H). One‐way ANOVA showed a time‐dependent decrease in C6 cell viability for both MZ formulations, starting at 250 μM after 1 h incubation [F_(11,121)_ = 316.5; *p* < 0.001] (Figure 1A,E), at 50 μM after 3 h [F_(11,130)_ = 184.8; *p* < 0.001] (Figure 1B,F), at 20 μM after 6 h [F_(11,143)_ = 415.5; *p* < 0.001] (Figure 1C,G), and at 10 μM after 24 h [F_(11,133)_ = 413.5; *p* < 0.001] (Figure 1D,H).

**FIGURE 2:**
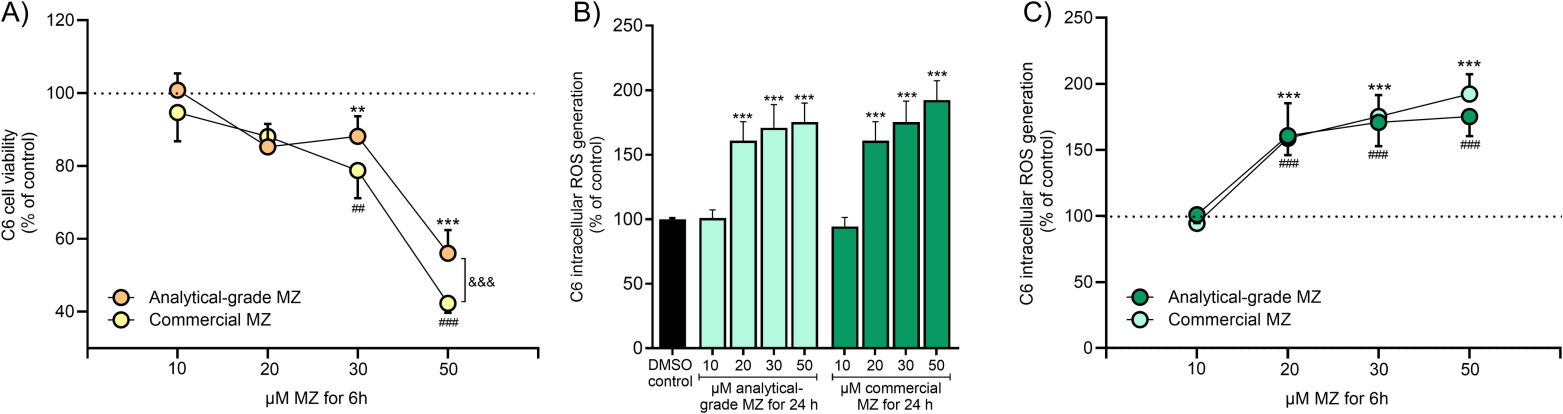
Comparative effects of commercial and analytical‐grade Mancozeb (MZ) on C6 cell viability and intracellular ROS generation. C6 cells were incubated with increasing concentrations (10–50 μM) of commercial or analytical‐grade MZ for 6 h to compare the cell viability (A) and intracellular ROS generation (B and C). Data are presented as mean ± SEM and expressed as a percentage of the control group. Panel B: ***p < 0.001 compared to the control group. Panels (A and/or C): **p < 0.01 and *** *p* < 0.001 analytical‐grade MZ compared to the control group; ^##^ *p* < 0.01 and ^###^ *p* < 0.001 commercial MZ compared to the control group; ^&&&^ *p* < 0.001 compared between formulations. Statistical comparisons relative to control were performed using one‐way ANOVA, whereas differences between formulations were assessed using two‐way ANOVA, both followed by Tukey's post hoc test.

Although a similar time‐dependent decrease in cell viability was observed for both formulations, we further compared the toxicity profiles of commercial and analytical‐grade MZ by evaluating cell viability after 6 h of exposure (Figure 2A). Two‐way ANOVA revealed a significant effect of formulation, dose, and their interaction, with the commercial formulation inducing a greater reduction in cell viability at 50 μM (~60%) compared to the analytical‐grade MZ (~40%), both relative to control (between groups: [F_(1,42)_ = 28.04; *p* < 0.001]; between doses: [F_(3,10)_ = 292.1; *p* < 0.001]; and interaction between groups and doses [F_(3,84)_ = 8.8; *p* < 0.001]).

Furthermore, to investigate whether the observed difference in cell viability between commercial and analytical‐grade MZ was associated with oxidative stress, intracellular ROS generation was measured following the same exposure conditions (Figure 2B,C). One‐way ANOVA showed a significant increase in intracellular ROS generation starting at 20 μM for both formulations compared to the control group [F_(8,102)_ = 81.25; *p* < 0.001] (Figure 2B). However, although a trend toward increased ROS generation was observed between the two MZ formulations, particularly at 50 μM, two‐way ANOVA revealed no statistically significant difference between formulations (*p* = 0.9882).

## 4 Discussion

The present study demonstrates that both commercial and analytical‐grade MZ induce time‐ and concentration‐dependent cytotoxic effects on the viability of C6 glial cells. Significant cytotoxicity was observed following exposure to 250 μM of MZ for 1 h, while progressively lower concentrations elicited toxic effects with increasing exposure time, reaching significance at 10 μM after 24 h. These findings indicate both acute and cumulative toxic effects of MZ under experimental conditions relevant to toxicological (Tsang and Trombetta 2007) and neuronal models (Domico et al. 2007), as well as more recent evidence from neuronal systems and in vivo models (Favarin et al. 2023; Martins et al. 2023; Montgomery et al. 2018; Paganotto Leandro et al. 2023; Saraiva et al. 2021).

Notably, the concentration range used in the present study is consistent with previously reported in vitro findings demonstrating mancozeb‐induced cytotoxicity and oxidative stress within the micromolar range. In human erythrocytes, exposure to 5–100‐μM MZ induced oxidative damage and impaired cellular function (Quds et al. 2023). In neuronal models, micromolar concentrations ranging from 0.1 to 16 μM were sufficient to induce marked neurotoxicity in human neuroblastoma SH‐SY5Y cells, including significant reductions in cell viability and increased ROS generation (Cuadros‐Buenaventura et al. 2026). Together, these findings support the biological relevance of the concentration range used in the present study.

The neurotoxicity of MZ has been largely attributed to Mn, one of its major toxicologically relevant degradation byproducts, which is highly relevant for neurotoxicological outcomes in humans (Costa‐Silva et al. 2018; Favarin et al. 2023; Tinkov et al. 2021; Tsang and Trombetta 2007). Although Mn is an essential trace element involved in several physiological processes, including antioxidant defense and cellular metabolism (Chen et al. 2018), excessive accumulation in the CNS has been associated with neurotoxicity, particularly targeting astrocytes and dopaminergic neurons (Rovetta et al. 2007; da Silva et al. 2022; Li et al. 2023). Although the molecular mechanisms underlying Mn‐induced toxicity in C6 cells are not fully elucidated, mitochondrial dysfunction and increased ROS production have been consistently implicated as central mechanisms of Mn‐related cytotoxicity in neural cells, given the role of ROS in redox signaling and in the regulation of mitochondria‐mediated apoptotic pathways (Redza‐Dutordoir and Averill‐Bates 2016). In line with this, da Silva et al. (2022) demonstrated that Mn exposure reduces C6 cell viability and induces inhibition of mitochondrial respiratory chain Complexes I and II activity, along with increased ROS levels. Together, these findings suggest that Mn contributes to MZ‐induced neurotoxicity in C6 cells, potentially through mitochondrial dysfunction and oxidative stress.

To further investigate formulation‐dependent toxicity relevant to applied toxicology, we directly compared the effects of the commercial formulation (Unizeb Gold) and analytical‐grade (Pestanal) MZ on C6 cell viability and intracellular ROS generation following 6 h of exposure to concentrations ranging from 10 to 50 μM, corresponding to short‐term exposure conditions. Although both formulations significantly reduced cell viability from 20 μM onwards, a formulation‐dependent effect emerged at higher concentrations. Specifically, at 50 μM, the commercial formulation induced a greater reduction in cell viability (~60%) compared with the analytical‐grade MZ (~40%), both relative to control, as supported by the significant interaction between formulation and dose identified in the two‐way ANOVA. Importantly, this effect was not observed across all concentrations but was restricted to higher concentrations, reinforcing the presence of a concentration‐dependent formulation effect.

Despite this difference in cytotoxicity, both formulations elicited comparable increases in intracellular ROS levels, indicating that oxidative stress likely represents a shared mechanism of toxicity in both formulations, primarily associated with Mn content (Costa‐Silva et al. 2018; da Silva et al. 2022; Li et al. 2023). The similarity in ROS generation indicates that the greater toxicity induced by the commercial formulation cannot be fully explained by oxidative stress alone under the conditions tested.

Importantly, this enhanced toxicity is unlikely to be attributable to Mn concentration alone and instead suggests an additional contribution of coformulants present in the commercial product. Although the commercial formulation contains 750 g/kg (75% w/w) of the active ingredient and approximately 25% coformulants, both formulations were prepared and tested based on equivalent nominal concentrations of MZ. Therefore, the observed differences in cytotoxicity cannot be explained by differences in active ingredient concentration, but rather indicate a role of coformulants present in the commercial product. Pesticide formulations are complex mixtures comprising declared active ingredients and various coformulant additives, many of which are classified as inert and protected as confidential business information (Defarge et al. 2016). According to product labeling, these components include dispersing and wetting agents, which may influence the biological effects of the formulation (UPL Brasil 2024).

In this context, toxicity may arise through multiple pathways. In addition to increasing membrane permeability through surfactant‐like activity, formulation excipients may also influence the physicochemical behavior of metal ions released from the mancozeb complex. Mancozeb contains both Mn and Zn coordinated to the dithiocarbamate ligand, and under biological conditions, these metals may dissociate and interact with cellular components. Recent studies suggest that formulation additives may alter the solubilization and release of cations derived from pesticide complexes, thereby influencing their bioavailability and biological interactions (Cuadros‐Buenaventura et al. 2026). In addition, the biochemical effects of extracellular metal ions such as Zn^2+^ depend strongly on their chemical speciation in the surrounding environment (Haase et al. 2015). Although zinc is not the primary focus of the present study, it is known that Zn^2+^ can interact with neuronal structural proteins and intracellular signaling pathways (Kress et al. 1981; Krężel and Maret 2021). Therefore, changes in metal ion availability mediated by formulation components may represent an additional mechanism contributing to the enhanced cytotoxicity observed for the commercial formulation.

Although regulatory toxicological assessments typically focus on isolated active ingredients, multiple studies have demonstrated that pesticide formulations can exert greater toxic effects than the active compounds alone (Benachour and Séralini 2009; Ferguson et al. 2022; Nagy et al. 2021; Nikoloff et al. 2013; Simasotchi et al. 2021). Regulatory agencies increasingly emphasize the need to assess the toxicological risks of all formulation components, including coformulants and adjuvants (Brasil 2002; United States Environmental Protection Agency 2024). Enhanced toxicity of commercial formulations has been attributed to synergistic or additive interactions between active ingredients and coformulants, potentially mediated by increased membrane permeability, enhanced cellular uptake, or altered metabolic activation (Mesnage et al. 2013; Defarge et al. 2016). Consequently, under realistic exposure scenarios, the combined effects of MZ formulations and their metabolites may pose greater toxicological risks than the active ingredient alone, potentially leading to an underestimation of formulation‐dependent hazards when assessments focus exclusively on isolated compounds (Jorge‐Escudero et al. 2022; Todt et al. 2016).

Taken together, our findings suggest that unidentified coformulants present in commercial MZ formulations may enhance cytotoxicity in this astrocyte‐like model through mechanisms beyond oxidative stress. Given the presence of dispersing and wetting agents, it is plausible that surfactant‐like compounds increase membrane permeability and facilitate cellular uptake, thereby amplifying cytotoxic responses. In addition, formulation components may influence the solubilization, release, and chemical speciation of metal ions derived from the MZ complex, thereby modifying their bioavailability and interactions with cellular targets. In this context, MZ‐derived metals such as Mn and Zn may become more bioavailable under specific physicochemical conditions created by formulation additives, which may enhance their reactivity and biological effects. Furthermore, degradation products associated with dithiocarbamate fungicides, such as ethylene thiourea (ETU), a well‐known toxic metabolite of ethylene bisdithiocarbamates, may also contribute to enhanced toxicity. Additionally, trace amounts of other metals present in the formulation may also contribute to altered cellular responses. These components, individually or synergistically, may promote redox imbalance, mitochondrial dysfunction, and activation of cell death pathways more robustly than the analytical‐grade MZ.

Finally, some limitations of this study should be acknowledged. The conclusions are based on experiments using a single glial cell line (C6 cells), which, despite its widespread use as an astrocyte‐like model, does not fully capture the cellular complexity of the mammalian CNS. Cell viability was assessed using the MTT reduction assay, which primarily reflects mitochondrial metabolic activity. Although this method is widely used and validated for cytotoxicity assessment, complementary viability assays could provide additional confirmation of the observed cytotoxic effects. In addition, although intracellular ROS generation was evaluated as an indicator of oxidative stress, the present study did not include complementary mechanistic endpoints related to mitochondrial dysfunction, redox imbalance, or cell death signaling. Therefore, the current findings should be interpreted as evidence of formulation‐dependent cytotoxicity associated with oxidative stress, rather than as a complete mechanistic characterization of neurotoxic pathways. Further, the undisclosed nature of coformulant composition and concentrations limited direct assessment of their individual toxicological contributions. In addition, the present study did not directly evaluate potential changes in metal ion speciation or cation release from the MZ complex in the presence of formulation additives. Future studies combining toxicological and analytical approaches will be necessary to better characterize how formulation components influence metal availability, evaluate their toxicity in isolation, and investigate these effects in vivo, thereby enhancing the translational relevance of these findings for applied and human toxicology.

## 5 Conclusion

The present study demonstrates that both commercial and analytical‐grade MZ induce time‐dependent cytotoxic effects in C6 glial cells, resulting in significant reductions in cell viability. Notably, the commercial formulation elicited a greater reduction in cell viability than the analytical‐grade MZ, despite inducing comparable increases in intracellular ROS levels. This dissociation between cytotoxicity and oxidative stress suggests that formulation components may enhance toxicity through mechanisms beyond oxidative stress alone, potentially by modifying the availability or biological interactions of metal ions derived from the MZ complex. These findings reinforce the importance of evaluating complete pesticide formulations, rather than isolated active ingredients, to achieve a more realistic assessment of toxicological risk relevant to human and occupational health. Future studies incorporating additional cellular models, in vivo approaches, and analytical characterization of formulation components will be essential to further elucidate the mechanisms underlying formulation‐dependent toxicity.

## Author Contributions

All authors contributed to the study conception and design. L. L. designed, planned, and performed the cellular experimental model, carried out biochemical measurements and data analyses and revised the manuscript. F.V. performed the cellular experimental model, wrote, and revised the manuscript. K.G.V. performed the cellular experimental model, carried out biochemical measurements and data analyses, and revised the manuscript. V.G. performed the cellular experimental model and carried out the biochemical measurements. D.C. performed the statistical analyses, wrote, and revised the manuscript. A.P.R. coordinated all experimental procedures and data analyses, prepared the final version of the figures, wrote the manuscript, was responsible for financial support acquisition, and provided the required infrastructure.

## Funding

This work was supported by grants from FAPESC (Fundação de Amparo a Pesquisa e Inovação do Estado de Santa Catarina) by Edital FAPESC n° 15/2023—Programa de Estruturação acadêmica para Latoratórios Multiusuários dedicados à pesquisa avançada no estado de Santa Catarina (Multilab).

## Ethics Statement

The authors have nothing to report.

## Consent

The authors have nothing to report.

## Conflicts of Interest

The authors declare no conflicts of interest.

## Data Availability

The datasets generated during and/or analyzed during the current study are available from the corresponding author on reasonable request.
